# Transthoracic ultrasound sign in severe asthmatic patients: a lack of “gliding sign” mimic pneumothorax

**DOI:** 10.1259/bjrcr.20190030

**Published:** 2019-11-15

**Authors:** Anna Del Colle, Giovanna Elisiana Carpagnano, Beatrice Feragalli, Maria Pia Foschino Barbaro, Donato Lacedonia, Giulia Scioscia, Carla Maria Irene Quarato, Enrico Buonamico, Maria Giulia Tinti, Gaetano Rea, Cristiana Cipriani, Elisabettamaria Frongillo, Salvatore De Cosmo, Giuseppe Guglielmi, Marco Sperandeo

**Affiliations:** 1 Institute of Respiratory Disease, Department of Medical and Surgical Sciences, University of Foggia, Foggia, Italy; 2 Department of Medical, Oral and Biotechnological Sciences, “G d’Annunzio”, Adriatic University, Chieti, Italy; 3 Department of Medical and Surgical Sciences, University of Foggia, Italy; 4 Institute of Respiratory Disease, Aldo Moro University of Bari, Bari, Italy; 5 Department of Internal Medicine IRCCS Fondazione Casa Sollievo della Sofferenza, San Giovanni Rotondo, Italy; 6 Department of Radiology, Ultrasound Diagnostic Unit, Monaldi Hospital, AO dei Colli, Naples, Italy; 7 Department of Internal Medicine and Medical Disciplines, “Sapienza” University of Rome, Italy; 8 Unit of Thoracic Surgery, IRCCS Fondazione Casa Sollievo della Sofferenza, San Giovanni Rotondo, Italy; 9 Institute of Radiology Department of Medical and Surgical Sciences, University of Foggia, Foggia, Italy; 10 Unit of Interventional and Diagnostic Ultrasound of Internal Medicine IRCCS Fondazione Casa Sollievo della Sofferenza, San Giovanni Rotondo, Italy

## Abstract

Transthoracic ultrasound (TUS) is a validate complementary technique widely used in everyday medical practice. TUS is the gold-standard for studying pleural effusion and for echo-guided thoracentesis, moreover, it is employed in detection of pleural and pulmonary lesions adherent to pleural surface and their ccho-guided percutaneous needle biopsy (PTNB).^1^ We used TUS technique to study severe asthma patients. We found that several patterns are constant in these patients. One of these patterns, *i.e*. lack of gliding sign, mimic pneumothorax (PNX). In this study, we attempted an echographic approach to asthma, trying to lay the first stone for the individuation of common ultrasound patterns in this disease.

## Background

Asthma is an important cause of morbility and mortality worldwide and it can affect people of any age. It is a common chronic airway disease, mainly marked by bronchial inflammation, bronchial hyperresponsiveness and variable expiratory airflow limitation that may resolve spontaneously or in response to medication. Clinically, asthma is defined by a history of respiratory symptoms such as wheeze, shortness of breath, chest tightness and cough. Symptoms can vary over the time and may sometimes be absent for weeks or months at a time.

We focused our attention on severe asthma (SA) by examining some severe asthmatic patients who usually come to our SA Centre for specialist examinations and follow-up. All our patients met the ERS/ATS criteria of SA,^2^ as they all were treated according to GINA^3^ Step 5 level of therapy, plus a biological drug (Omalizumab, Mepolizumab or Benralizumab) to obtain asthma control. We decided to study this kind of patients with transthoracicultrasound (TUS) technique, with the aim of identifying a typical echografic pattern of asthma.

Pleuropulmonary ultrasound findings in normal subjects are mainly generated by physical ultrasound effects, namely imaging artifacts. Indeed, normal lung anatomy is not visible on ultrasound because at the level of pleural cavity, air into the lungs reflects almost all of the ultrasound beam (more than 95 %) as a result of the great difference in acoustic impedance between the soft tissues (chest wall) and pulmonary air at this point. This interface generates not only the so-called “pleural line,” a visible hyperechoic line that comes and goes with respiratory excursions (the gliding or sliding sign), but also reverberation artifacts, vertical (“ring down” or B-lines) and horizontal (simple reverberations or A-lines). Under normal conditions, with a 3.5–5 MHz convex probe, the hyperechoic “pleural line” is visible as a white band of average thickness of 2 mm (range 1.4–2.8 mm). When an 8–12.5 MHz linear probe is used, the visible thickness of the hyperechoic “pleural line” is 1 mm (range 0.6–1.8 mm). The thickness of the “pleural line” can also vary according to the change in frequency of the probe, the presence or absence of the tissue harmonic and its focusing, as well as to the use of the probe (Linear, convex, phased array). It’s extremely important to know how the Ultrasound machine is set for thoracic imaging to avoid imaging misinterpretation: for example, a too high TGC (time gain compensation) generates artifacts which could lead to easy mistakes for the untrained eye.^4^ In effect, it is mandatory to remember that pleural line’s thickness is not anatomically related to parietal or visceral pleura or even to the pleural space, whose thickness does not exceed 150 µm.^4–6^

The thoracic cage and the scapular bones reduce the visible pleural surface: thus, TUS could detect pleural thickening, pleural/subpleural nodules, and other subpleural lung abnormalities only across this 70% of the subpleural surface that is visible to ultrasound.^4^

In healthy subjects, lung movement due to respiration is revealed by the lung “gliding sign” or “sliding sign,” and could be confirmed in M-mode by the presence of the “sea-shore sign,” which is the interface produced by the horizontal lines representing the motionless parietal layers and a granular pattern generated by the sliding lung.

Previous studies^7,8^ have shown that in another widespread obstructive disease, COPD, was detectable a lack of gliding sign and it could be due to a condition of lung hyperinflation, probably responsible of a reduction in respiratory movement and so of the pleural gliding sign. Hyperinflation, defined as an increased volume of air remaining in the lung at the end of spontaneous expiration, in asthma is a consequence of airway inflammation and bronchial wall thickening. Some causes of wall thickening such as airways wall edema, mucoid impaction, and bronchoconstriction are potentially reversible. Subsequently, chronic airway inflammation, tissue injury, and following abnormal repair lead to irreversible structural changes in the airway walls of both asthmatics and COPD patients collectively referred to as “airway remodeling.” Airway remodelling consists in structural changes within the airway wall, such as an increased epithelium basal membranous thickness, hypertrophy of the smooth muscle cell, and peribronchial fibrosis. Indeed, postmortem examination of people died for fatal asthma consequences showed how this kind of patients have overinflated lungs, mucus plugs, damaged bronchial mucosae, submucosal edema, inflammatory cells infiltration, mucus glands hypertrophy, smooth muscle hyperplasia and basament membrane thickening.^9^

The presence of gliding sign is traditionally used for the exclusion of pneumothorax. Indeed, in pneumothorax diagnosis, ultrasound negative predictive value is almost 99%^10^ because the presence of gliding sign in B-mode and of the sea-shore sign in M-mode would exclude the pneumothorax, while ultrasound positive predictive value in detecting PNX is lower because there are also many other pathological conditions which mimic its ultrasound features.^4,11^ In particular, pathological condition characterized by air trapping and lung hyperinflation, like bullous emphysema,^7,12^ were found marked by the absence of gliding sign and so considered as TUS “false positive” of pneumothorax. On this background, we tried to understand if severe asthmatic patients could show echografic signs similar to PNX that correlates with a condition of air-trapping and hyperinflation, like in COPD and other pulmonary diseases. This kind of findings could advice against the use of TUS in severe asthmatic patients after thoracic invasive procedures in suspicion of pneumothorax, or during their follow-up, considering also that their condition could be characterized by symptoms that mimic those of pneumothorax, like chest pain and dyspnea,^13^ especially during severe exacerbations.

For this purpose we performed a complete TUS examination of 3 SA patients who were in follow up at our SA Centre at the time of this study. The severity of asthma was assessed by symptoms evaluation through asthma control test (ACT), spirometry results and the history of patients’ exacerbations, following ERS/ATS guidelines^2^ and GINA^3^ definitions. All the patients were under treatment following GINA Step 5 level of therapy including biological drugs (Omalizumab, Mepolizumab or Benralizumab).

The ultrasound investigation was carried out using an ultrasound scanner “Esaote My Lab 30” (Genova, Italy) with thoracic set up and with a convex probe (3.5–5 MHz) and a linear probe (8–12 MHz). A tissue harmonic focused on the pleural line was used, in an effort to reduce the natural artifacts, and the time gain compensation (TGC) not exceed the 50% of the total gain. We started the exploration of each hemithorax with the patient in a sitting position from the back, with longitudinal and transversal intercostal and paravertebral scans, exploring from the base up to the ipsilateral posterior pulmonary apex, passing, then, to the examination of the lateral chest side along the posterior, middle and anterior axillary line. The anterior chest has been evaluated with longitudinal and transversal intercostals and parasternal scans, with the addition of subxyphoid and supraclavicular views.

We compared our TUS findings with patients’ high resolution CT (HRCT). Indeed, HRCT is the gold-standard to investigate the morphological changes of the lung and bronchi*,* providing quantitative morphometry of airways and distal lungs and highlighting airway wall thickness,^2^ and it helps to identify lung abnormalities due to asthma, as bronchiectasis, bronchial wall thickening and morphological anomalies.^14^

Supplementary Video 1 Videoclip of TUS scans were viewed by two expert sonographers, in a double-blind way; also HRCT imaging were checked by two expert radiologist, in a double-blind way as well.

As far as we know, this is the first attempt to study this kind of patients by TUS in a systematic way, and we hope that our findings could be a starting point to study better strategies to study and follow-up asthmatic population.

## FIRST CASE

### Case presentation with figures

A 59-years-old, non-smoker, male, affected by severe, late-onset asthma, in periodic follow-up at our regional referring centre for Severe Asthma.

Uncontrolled by GINA^3^ step five therapy for asthma with high dose ICS (Beclometasone 400 mcg/twice a day), LABA (Formoterol 12 mcg/twice a day), LAMA (Tiotropium 5 mcg per day), LTRA (montelukast 10 mcg per day) and Benralizumab, after two failed attempt with Omalizumab and Mepolizumab; Spirometry (07/01/2019: FEV1 1,37 L- 41% - FVC 4.05 L - 96% - FEV1/FVC 34–44%) was performed and Asthma Control Test^14^ was administered. The patient reported more than four exhacerbations/year.

### Discussion

**Figure 1.**
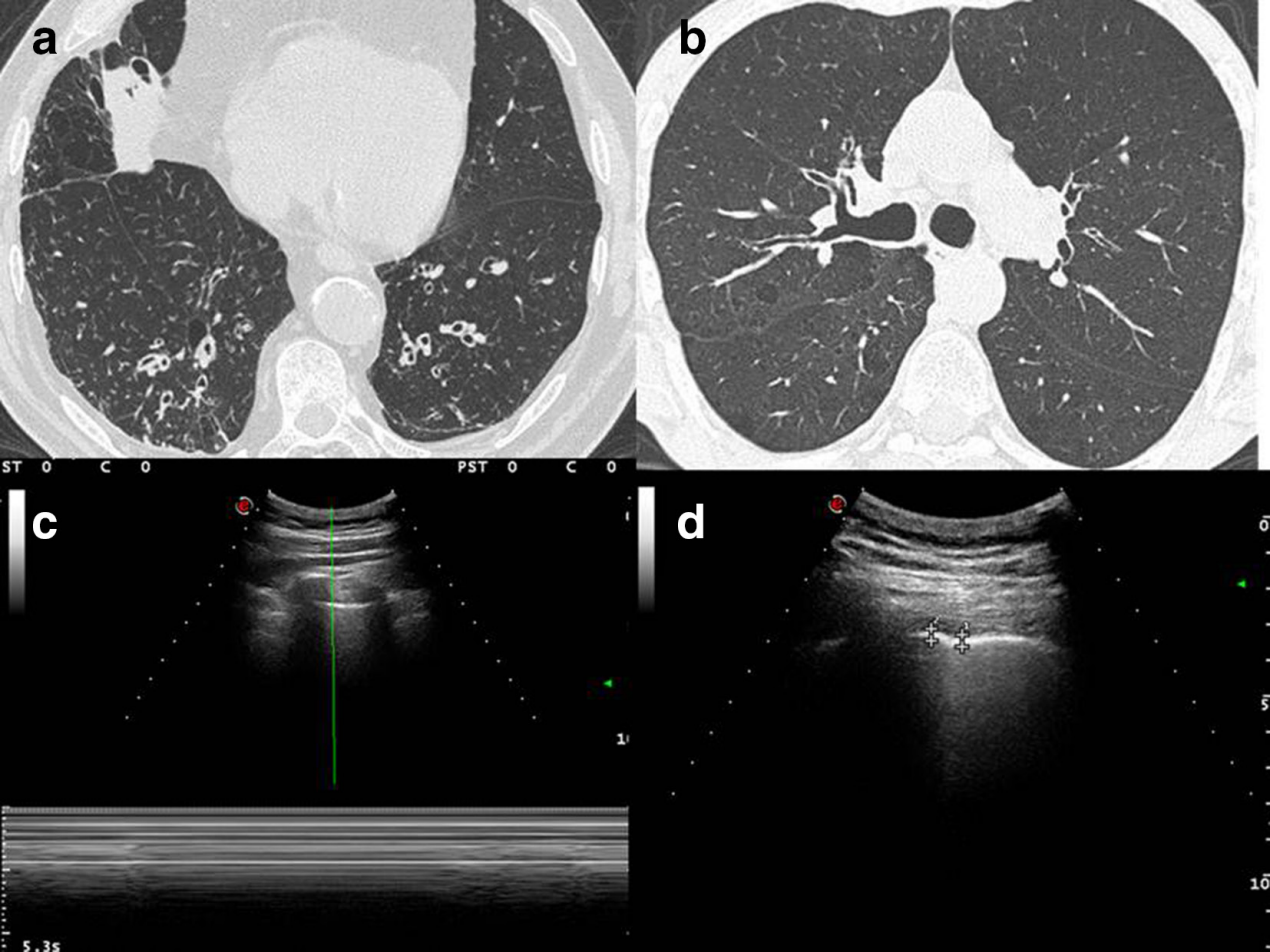
(A, B) HRCT imaging shows overdistension and the airway wall thickening with bronchiectasis of lung bases; in addition, it shows also centrilobular micronodular lesions which state distal airway inflammation. (C) Ultrasound imaging, taken with convex probe (5 MHz), indicates the absence of gliding sign, which was confirmed by Barcode sign in M-mode. (D) Ultrasound imaging, with convex probe (5 MHz) shows an irregular thickening of the hyperechoic pleural line (3.1–3.6 mm). HRCT, high resolution CT.

HRCT imaging (Figure 1A, B) corroborates overdistension and the airway wall thickening with bronchiectasis of lung bases; in addition, it shows also centrilobular micronodular lesions which state distal airway inflammation.

A complete TUS exam was performed by an expert clinician and we compared it with a CT of the patient (nowadays HRCT is the gold standard for studying lung diseases).

Ultrasound imaging, taken with convex probe (5 MHz), indicate the absence of gliding sign, which was confirmed by Barcode sign in M-mode (Figure 1C) and an irregular thickening of the hyperechoic pleural line (3.1–3.6 mm, where the normal value is under 3 mm) (Figure 1D). All these findings were present anteriorly and in middle and in apexes posteriorly, bilaterally in both the chest walls. No evidence of pleural effusion.

### Learning points

- Irregular thickening of pleural line, detected by convex probe (5 MHz, thickening >3 mm) and linear probe (10 MHz, thickening >2 mm).

- absence of gliding sign and presence of Barcode sign in M-mode, presence of lung point.

## Second case

### Case presentation with figures

A 59-years-old, non-smoker, male, affected by severe allergic asthma with bronchiectasis, in periodic follow-up at our regional referring centre for SA.

Controlled by GINA^3^ step five therapy for asthma with high dose ICS (Beclometasone 400 mcg/twice a day), LABA (Formoterol 12 mcg/twice a day), LAMA (Tiotropium 5 mcg per day), LTRA (montelukast 10 mcg per day) and Omalizumab; Spirometry (FEV1 2,69–77%: FVC 4,09–92%; FEV1/FVC 66–84%) was performed and ACT (24) was administered. The patient did not report any exhacerbation during the last year.

### Discussion

**Figure 2.**
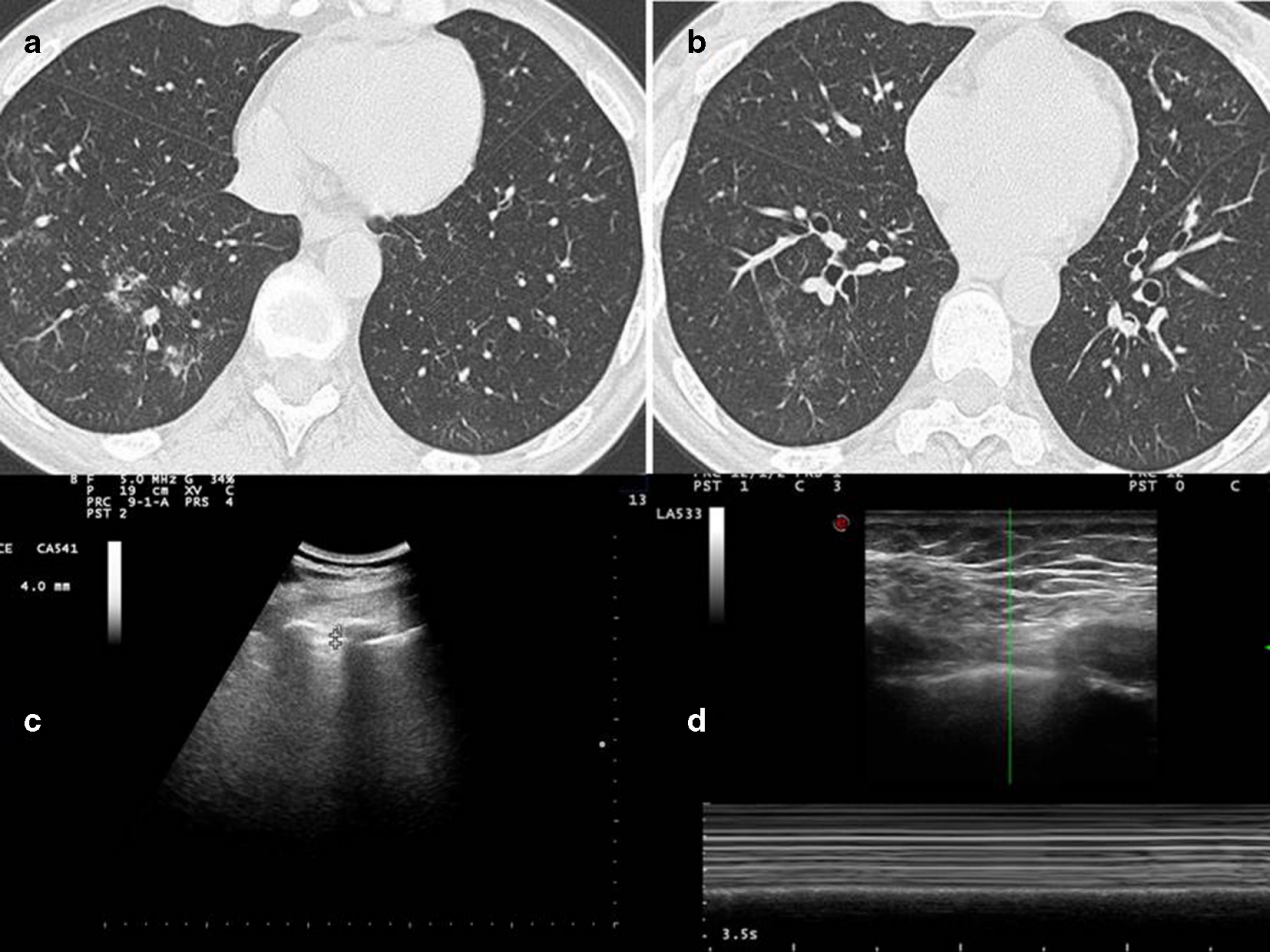
(A) HRCT imaging shows bronchial wall thickening, endobronchial nodules in segmental and subsegmentary bronchi of the lower left lobe. (B.) The more caudal scan shows the presence of focal areas of ground glass hyperdensity in the peribronchiolar area in the basal pyramid of the lower right lobe, in relation to flogistic alterations of distal airways. (C) Ultrasound imaging with convex probe (5 MHz) shows an irregular, limited but multiple focal thickening of the hyperechoic pleural line (3.2–4 mm) (D). the lack of gliding sign is confirmed by Barcode sign in M-mode with linear probe (10 MHz) (Figure D2). HRCT, high resolution CT.

HRCT imaging shows bronchial wall thickening, endobronchial nodules in segmental and subsegmentary bronchi of the lower left lobe (Figure 2A). The more caudal scan (Figure 2B) shows the presence of focal areas of ground glass hyperdensity in the peribronchiolar area in the basal pyramid of the lower right lobe, in relation to flogistic alterations of distal airways.

A complete TUS exam were performed by an expert clinician and then compared with a CT of the patient. Using convex probe (5 MHz), we found an irregular, limited but multiple focal thickening of the hyperechoic pleural line (3.2–4 mm, where the normal values are under 3 mm) anterior and posterior, bilaterally (Figure 2C). Using linear probe (10 MHz), we found that the gliding sign was absent in middle posterior and the apexes, bilaterally, and this finding was confirmed by Barcode sign in M-mode (Figure 2D). There was no evidence of pleural effusion.

### Learning points

- Irregular thickening of pleural line, detected by convex probe (5 MHz, thickening >3 mm) and linear probe (10 MHz, thickening >2 mm).
- Absence of gliding sign and presence of Barcode in M mode, presence of lung point.

## Third case

### Case presentation with figures

A 61-years-old, non-smoker, female, affected by late-onset eosinophilic severe asthma, in periodic follow-up at our regional referring centre for SA.

Uncontrolled by GINA^3^ step five therapy for asthma with high dose ICS (Beclometasone 400 mcg/twice a day), LABA (Formoterol 12 mcg/twice a day), LTRA (montelukast 10 mcg per day) and Mepolizumab; Spirometry (FEV1 0.75–42%; FVC 0.97–45%; FEV1/FVC 77–99%) was performed and ACT^15^ was administered. The patient reported more than four exhacerbations/year.

### Discussion

**Figure 3.**
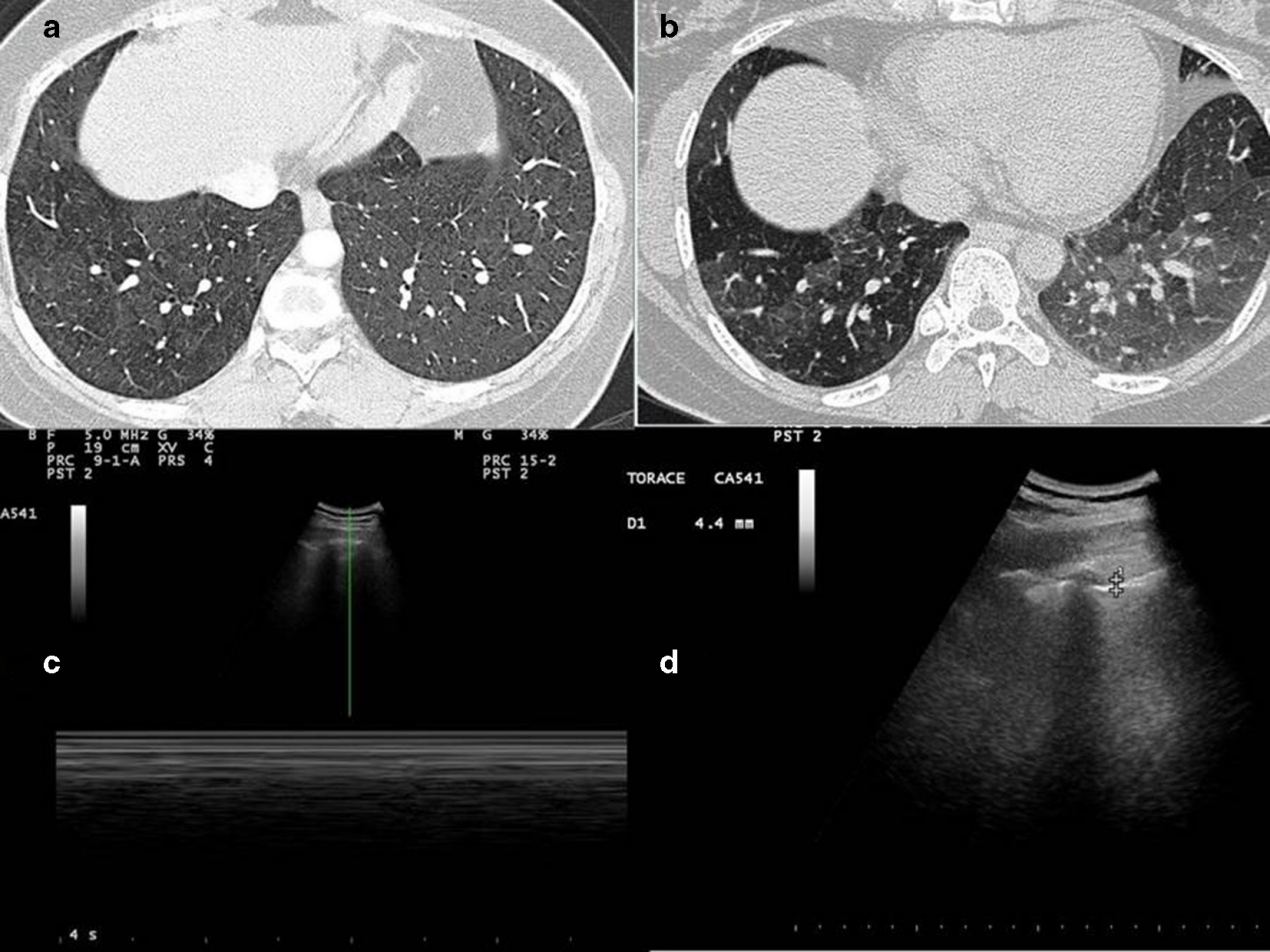
(A, B) HRCT scans show minimal thickening of the bronchial walls and a slightly inhomogeneous appearance of the lung parenchyma (a mild picture of “mosaic oligohemia”). (C) Lack of gliding sign is confirmed by Barcode sign in M-mode with convex probe (5 MHz). (D) Ultrasound imaging with convex probe (5 MHz) shows an irregular thickening of the hyperechoic pleural line (3.1–4.4 mm). HRCT, high resolution CT.

HRCT scans performed in inspiratory apnea (Figure 3A and B) demonstrate minimal thickening of the bronchial walls and a slightly inhomogeneous appearance of the lung parenchyma (a mild picture of “mosaic oligohemia”).

A complete TUS exam was performed by an expert clinician and we compared it with a CT of the patient. The gliding sign was absent in middle and the apexes, anteriorly and posteriorly, and this finding was confirmed by Barcode sign in M-mode (**Figure 3C**). Ultrasound imaging, taken with convex probe (5 MHz), shows an irregular thickening of the hyperechoic pleural line (3.1–4.4 mm) in anterior and posterior scan, bilaterally in both the chest walls (Figure 3D).There was not pleural effusion.

### Learning points

- Irregular thickening of pleural line, detected by convex probe (5 MHz, thickening >3 mm) and linear probe (10 MHz, thickening >2 mm).
- Absence of gliding sign and presence of Barcode in M mode, presence of lung point.

## Discussion and conclusions

In all the patients we studied with TUS, the main signs observed were the lack of “gliding sign” in medial posterior and anterior thoracic scan, with a “Barcode” sign in M-mode. These findings could be due to a condition of lung hyperinflation at this level as a result of chronic inflammation, bronchial hyperresponsiveness and narrowing secondary to airways remodeling. The increased volume of air remaining in the lung at the end of spontaneous expiration is probably responsible of a reduction in respiratory movement as in the pleural gliding sign. We believe that these findings are extremely interesting because the absence of “gliding sign” and the Barcode sign in M-mode that we found in all our patients with severe asthma are traditionally signaled as a typical feature of pneumothorax. In addition, we found in our patient also the “lung point,” a dynamic ultrasound sign which represent the sudden change between the part of normal pleura with the presence of gliding sign and the pathological part with the lack of gliding sign and is another ultrasound sign usually considered as pathognomonic of pneumothorax. So, we may have identified an ultrasound false positive of PNX. Indeed, other conditions marked by a mobility reduction of pleuras and lungs are reported in literature to reproduce ultrasound signs of PNX: fibrothorax, panlobular emphysema, severe fibrosis with honeycombing and traction cysts, pleural adhesion, loculated pleural effusions, obstructive atelectasis, abscesses, empyema, atelectasis, cysts, blebs, lung contusions, tumors infiltrating the chest wall.^4,8,11,16,17^ Also in COPD, another widespread obstructive disease, previous studies showed a lack of gliding sign.^7^ In our opinion, severe asthma can be correctly inserted in the list of false-positive conditions of pneumothorax, being severe asthma, like COPD, a condition characterized by lung hyperinflation mainly due to the extension of chronic inflammation and subsequent remodeling processes to peripheral airways. Thus, a lack of the “gliding sign” is common to many conditions and not specific of pneumothorax. All false-positive conditions of PNX are common comorbidities in patients with acute respiratory symptoms who usually require a visit in emergency room. Therefore, it could be useful to know that there are a lot of conditions which can mimic a PNX especially in an emergency setting where an erroneous management due to a misdiagnosis based on ultrasound pattern only can lead to fatal consequences (*i.e.* the insertion of a chest tube in a big emphysematous bulla).^15^ On this regard, we should consider that also an asthmatic crisis in mild asthma can mimic symptoms (dyspnea, chest pain etc.) and ultrasound signs of pneumothorax, being the asthmatic crisis itself characterized by a condition of dynamic hyperinflation. Therefore, in patient with underlying diseases, particularly chronic obstructive pulmonary disease, TUS is not an accurate and sensitive technique to detect a pneumothorax certainly^18,19^ and physicians have to pay close attention to not emphasize the substitutive role of a method, such as TUS, considered by international guidelines useful but only complementary to the other radiological diagnostic investigations (*i.e.* chest X-ray or chest CT scan, that is the gold-standard) in the diagnosis of PNX. Moreover, we identified in all our patients an irregular thickening of hyperechoic pleural line, especially in medial-basal posterior ultrasound scans. Comparing with HRCT imaging, we noticed that the pleural thickening correlates with subpleural areas marked by distal airways inflammation.

Peripheral airways have been recognized as a predominant site of inflammation, airflow obstruction and remodeling in severe asthmatics. This aspect could be interesting because performing an HRCT can provide useful information in SA patients, especially in those with a particular phenotype (*i.e.* asthma with fixed airway obstruction characterized by airways remodeling and rapid decline in lung function). Indeed, in this type of patients is also suggested by ERS/ATS guidelines**^2^** to perform a chest HRCT, but this kind of radiological exam has several potential complications and costs, thus it cannot be used as a routine test for follow-up. On the contrary, TUS is a rapid, safe, low cost, easy to use and easy to have exam in every department of all hospitals; in addition, there is no ionizing radiation exposure issue with thoracic ultrasound.^20^ In this context, we may have identified a pattern of ultrasound signs which could be used in follow up of severe asthmatic patients and which has a correspondence with HRCT imaging. A limit to underline of the ultrasound examination in the follow up of asthmatic patients could be the lack in the exact morphological correlation between the transthoracic ultrasound signs (expressed substantially as artifacts) and the underlying pathology (hyperinflation of the peripheral airways) that affect its reproducibility. Thus, further prospective studies with a wider range of cases, as the one we are working on, are needed to confirm this ultrasound pattern in SA patients and to see if these findings could have a practical value in follow-up; in the latter case, TUS could be a useful examination in the follow up of these patients during periodic medical check up in a SA centre, where an expert sonographer should perform periodically this exam.

### Videoclip Legends

Ultrasound middle-back posterior right scan in patient with severe asthma with linear probe (12.5 MHz): the absence of pleural line movement is highlighted by the lack in gliding sign in B-mode and, immediately after, by the bar code sign using M-mode.
